# A case report of allergic eczematoid dermatitis around hemodialysis access due to iodine-containing disinfectant

**DOI:** 10.3389/fimmu.2025.1627179

**Published:** 2025-07-28

**Authors:** Xiaomei Lu, Jing He, Liansheng Ren, Rong Yang, Shuhuan Liu, Jingying Bai, Yueqiu Tang, Yi Zeng, Yunmin Wang, Wei Zhang, Dan Zhu

**Affiliations:** ^1^ Department of Endocrine Nephrology, 363 Hospital, Chengdu, Sichuan, China; ^2^ Center for Endemic Disease Control, Chinese Center for Disease Control and Prevention, Harbin Medical University, National Health Commission & Education Bureau of Heilongjiang Province, Key Laboratory of Etiology and Epidemiology, Harbin Medical University, Center for Chronic Disease Prevention and Control, Harbin Medical University, Harbin, China

**Keywords:** hemodialysis, arteriovenous fistula, allergic eczematoid dermatitis, iodine-containing disinfectant, tunneled cuffed catheter

## Abstract

We report here a case of allergic eczematoid dermatitis related to hemodialysis access. The patient was initially suspected to have puncture needle allergies or dialyzer reactions until the patient develops a similar response after switching from an arteriovenous fistula (AVF) to a tunneled cuffed catheter (TCC) for dialysis. Thereby, we highly suspected that the allergen was the iodine-containing disinfectant used prior to dialysis, and the patient was ultimately diagnosed with iodine-containing disinfectant allergy. The patient’s dermatitis improved remarkably after switching to alcohol disinfection as well as taking oral steroids. Early identification and diagnosis of allergic reactions at the vascular access site can avoid contacting with allergens, accordingly prevent complications like infection and loss of precious vascular accesses in these patients.

## Introduction

Hemodialysis access is the lifeline for MHD patients. Current KDOQI guideline recommend that AVF should be the first choice for long-term vascular access owing to its advantage on TCC (1). Both types of accesses may fail due to thrombosis, infection, etc (2–4). The utilization of iodine-containing disinfectant on the skin around the fistula or catheter exit site before hemodialysis can reduce the risk of infections (5, 6). However, it is relatively rare for iodine-containing disinfectant to cause allergic reactions that lead to eczematoid skin lesions around the hemodialysis access, ultimately resulting in the discontinuation of the access. Here, we report a case of allergic eczematoid dermatitis triggered by iodine-containing disinfectant as an allergic factor and summarize the treatment experience. This serves as a crucial reminder that monitoring adverse reactions during the utilization of disinfectant for hemodialysis access. Severe allergic eczematoid dermatitis induced by iodine-containing disinfectant can lead to the discontinuation of the access, may threaten the lives of MHD patients.

## Case report

**Figure 1:**
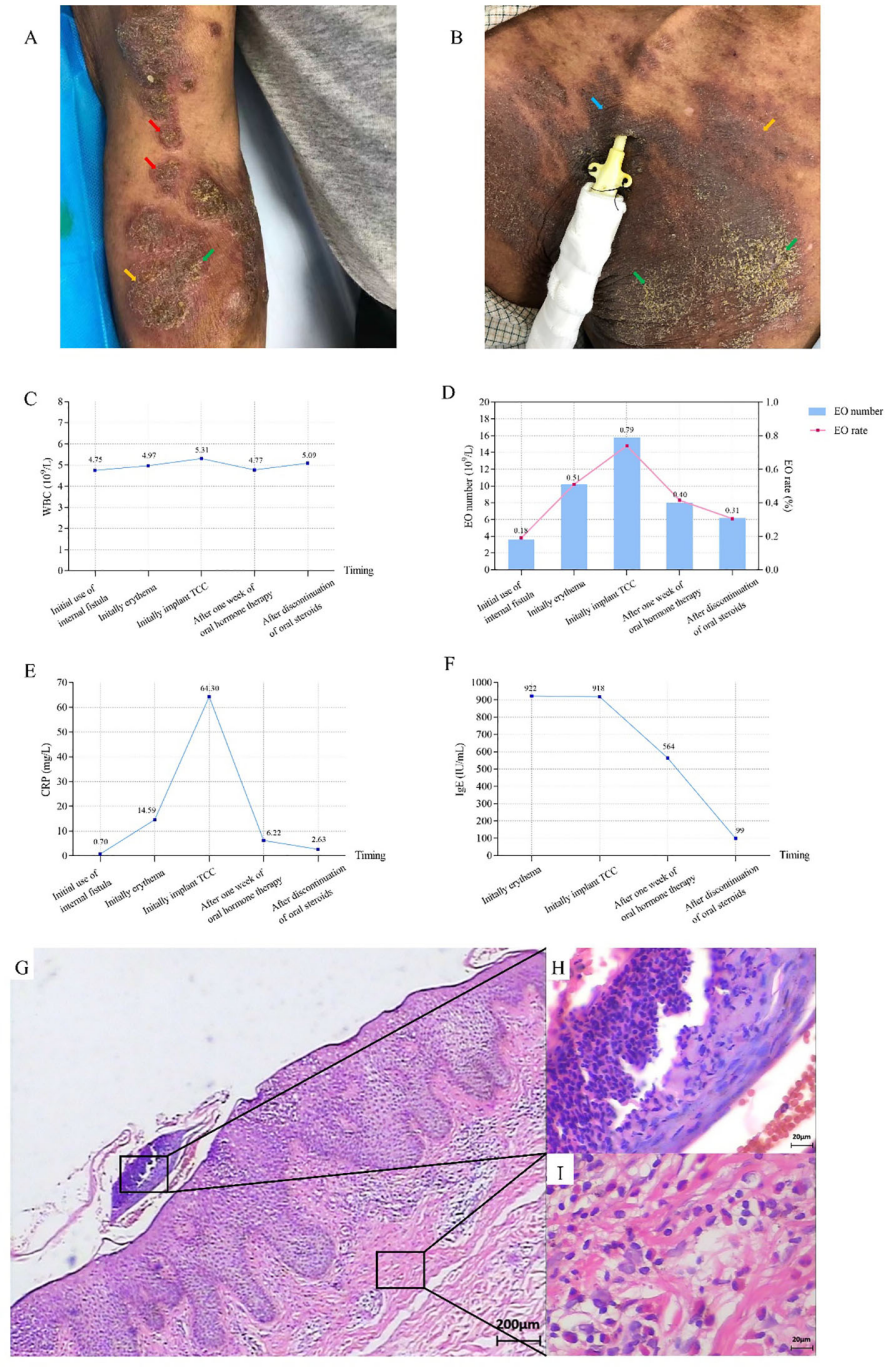
Photos of the patient’s lesion site, pathological pictures and inflammatory indicators before treatment **(A)** Skin lesions surrounding the AVF puncture site: redness (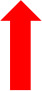), eczema (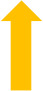), and scales (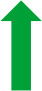). **(B)** The pigmentation (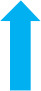), eczema, and scales in the sterilized area of TCC. **(C–F)** Changes of inflammatory indicators during different periods. WBC: white blood cell, EO: Eosinophil, CRP: C-reactive protein, IgE: immunoglobulin E. **(G–I)** Pathological damage was analyzed by H&E staining. **(G)** HE staining of the superficial dermis (×40). **(H)** Eosinophilic infiltration of the superficial dermis (×400). **(I)** Lymphocyte, plasma cell infiltration of the superficial dermis (×400).

**Figure 2:**
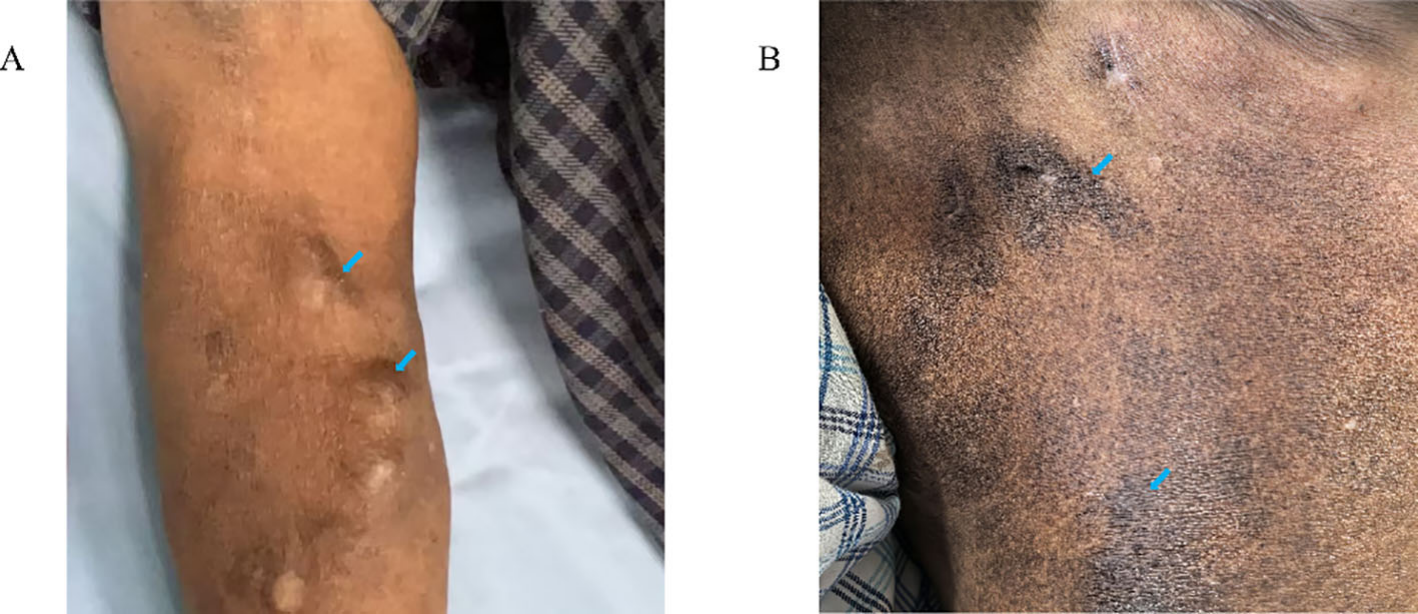
Photo of the patient’s lesion site after treatment **(A, B)** Hyperpigmentation (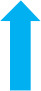) persisted in the disinfected areas of AVF **(A)** and TCC **(B)**.

Mr. Ren is an 83-year-old male with more than 4 years of dialysis age. He has a long-standing history of hypertension, and began hemodialysis treatment after right femoral vein catheter placement. He also underwent right forearm AVF surgery, and after the fistula matured, he has been using the fistula for long-term dialysis. The redness, eczema and scales gradually appeared on his AVF arm after 3 months. The dermatological diagnosis was allergic eczematoid dermatitis. Treatment with fusidic acid and oral antihistamines showed no significant improvement while applying topical halometasone relieve symptoms. Even worse, the lesions recurred repeatedly, with local pigmentation and lichenification. Subsequently, the skin temperature rose and local swelling became pronounced (shown in **Figure 1A**), further enhancing the risk of infection. Unfortunately, since the patient had consulted multiple departments in several hospitals before admission and had already used topical and oral antibiotics, bacterial smear and culture tests were not completed. We implanted a TCC in the patient’s right internal jugular vein, without skin lesions. However, one month later, similar lesions appeared around the catheter (shown in **Figure 1B**), with inflammatory indicators significantly increased, especially for immunoglobulin E (IgE) and C-reactive protein (CRP) (shown in **Figures 1C–F**). The biopsy of the skin around the catheter indicated epidermal hyperplasia, elongation of epidermal protrusions, and the presence of neutrophils and eosinophils in the epidermis. Additionally, there were lymphocytes, plasma cells, and eosinophil infiltration in the superficial dermis (shown in **Figures 1G–I**). Special staining of the skin biopsy specimen demonstrated a negative fungal immunofluorescence signal. Moreover, patch testing showed positive for povidone-iodine and aneriodine disinfectant. Instead, the 75% alcohol test was negative. Accordingly, we switched to 75% alcohol for disinfection, and administered oral prednisone 15 mg daily and loratadine 10 mg daily for one month, we observed that the local lesions did not spread further, lichenification improved significantly, but pigmentation persisted (shown in **Figures 2A, B**). The TCC was removed, and the patient resumed long-term dialysis with the AVF. Collectively, the patient was considered to have allergic eczematoid dermatitis caused by iodine-containing disinfectant.

## Discussion

Allergic eczematoid dermatitis is a type of delayed hypersensitivity reaction (Type IV) characterized by eczematous skin inflammation resulting from contact with a specific allergen. Clinically, it presents with redness, papules, vesicles, itching, and scaling. In chronic stages, lichenification may occur (7). The condition is mediated by T lymphocytes sensitized to allergens such as metals (e.g., nickel), fragrances, or chemicals, leading to immune-mediated epidermal and dermal damage. It is often synonymous with allergic contact dermatitis and distinct from irritant or atopic dermatitis based on its immunologic mechanism and trigger specificity. It needs to be differentiated from irritant dermatitis. The patient began to experience symptoms three months after using the arteriovenous fistula, and there was no history of acute onset, therefore, the possibility of irritant dermatitis is considered low. In this case, after prolonged use of arteriovenous fistula and TCC dialysis, the patient developed redness, scaling, vesicles, and thickening of the skin in areas disinfected with iodine-containing disinfectant of povidone-iodine. Topical treatment received significant effects, but the condition recurred repeatedly and would gradually spread. Even secondary local infections occurred in the arm where the arteriovenous fistula was located. Switching to alcohol for disinfection halted further progression of the skin lesions. Based on the patient’s typical clinical presentation and laboratory findings, the diagnosis of allergic eczematoid dermatitis is eventually confirmed, with iodine-containing disinfectant identified as a local sensitizing factor.

Although the initial irritation is often considered minor, it indicates that prolonged exposure can lead to more severe reactions, with the local lesions continuously spreading with ongoing exposure.

During hemodialysis maintenance, common allergens include disinfectant, puncture needles, and dialysis-related catheters. A prospective study showed that 25% of patients are sensitized to at least one dialysis-related allergen, and 8% of hemodialysis patients have active eczema around the AVF (8). Yang et al. reported a case of AVF-related eczema caused by a puncture needle as an allergen, while Batta et al. reported eczema caused by chlorhexidine sensitization (9, 10). Although allergic eczematoid dermatitis is frequent in dermatology, skin lesions related to hemodialysis access routes are relatively rare, and often misdiagnosed as general skin infections around the access sites. Early identification of povidone-iodine as a long-term sensitizer and its early discontinuation will prevent the severe progression of the AVF lesions. Repeated exposure to allergens at the AVF puncture site and around the TCC led to allergic reactions and expanding skin damage.

The treatment principle for this condition is to identify and remove allergens, avoid external irritants, and use oral antihistamines and topical medications. Long-term use of oral steroids is not recommended. However, due to the severity of the local lesions in this patient, and had irregularly and non-compliantly applied corticosteroid creams locally during the course of the disease, whether there was hormone-dependent dermatitis at the lesion site was unclear at that time, thereby a short-term oral course of 15 mg of prednisone was used with significant effect. This highlights the importance of symptomatic treatment as well as identifying allergens, both of which determine the severity of local lesions. We have also customized a long-term management plan for this patient: 1. Permanently prohibit the use of iodine-containing disinfectants. 2. Use 75% alcohol for long-term skin disinfection. 3. Educate the patient to recognize symptoms of allergic reactions. 4.Regularly monitor the skin condition around the vascular access site.

Maintaining a good skin condition around the vascular access is crucial for the smooth progression of dialysis. Timely identification of causes of skin damage is of great significance for the maintenance of the dialysis access. This case is relatively rare and has important practical implications for the treatment of hemodialysis patients. Inevitably, this study still has certain limitations due to the absence of post-treatment skin biopsy in the patient.

In conclusion, our unique case illustrates the importance in pinpointing the exact cause of allergic reactions in dialysis patients. The nephrologists should be more aware of all possible allergens in dialysis. Primary prevention involves the practical management of pre-dialysis procedures, such as using the minimum dose of iodine-containing disinfectant around the patient’s vascular access to observe for allergic reactions, thereby selecting the appropriate disinfectant.

## Data Availability

The raw data supporting the conclusions of this article will be made available by the authors, without undue reservation.
